# Is Moderately Hypofractionated Radiotherapy a Safe and Effective Strategy for Cervical Cancer?—A Review of Current Evidence

**DOI:** 10.3390/curroncol33010024

**Published:** 2026-01-01

**Authors:** Hui Xiao, Fuxin Guo, Zhenyu Wang, Kangjia Pei, Shuhua Wei, Ang Qu, Junjie Wang, Ping Jiang

**Affiliations:** Department of Radiation Oncology, Peking University Third Hospital, Beijing 100191, China; 2311210484@stu.pku.edu.cn (H.X.); wohewangayi@163.com (F.G.); 1810301343@bjmu.edu.cn (Z.W.); 2511210478@bjmu.edu.cn (K.P.); weishuhua@pku.edu.cn (S.W.); 0463180457@bjmu.edu.cn (A.Q.); junjiewang@pku.edu.cn (J.W.)

**Keywords:** cervical cancer, moderately hypofractionated radiotherapy, clinical outcomes, low- and middle-income countries

## Abstract

Cervical cancer (CC) remains a leading cause of cancer-related mortality, particularly in low- and middle-income countries (LMICs), despite advancements in HPV vaccination and screening. Radiotherapy (RT) plays a critical role in managing CC, but conventional fractionated radiotherapy (CFRT) is limited by long treatment durations, which reduce patient adherence, increase the risk of treatment interruptions, and impair healthcare access in LMICs. Moderately hypofractionated radiotherapy (MHRT) may offer a promising alternative, delivering higher doses per fraction with fewer total fractions, thus shortening treatment duration and alleviating the burden on both patients and healthcare systems. Early clinical data suggest that MHRT achieve acceptable short- to medium-term tumor control with manageable toxicity. However, the small sample sizes and limited follow-up in published studies preclude definitive conclusions about long-term efficacy and safety. This review synthesizes the existing clinical evidence to outline the potential benefits and inherent limitations of MHRT in CC management and highlight the need for future large-scale, long-term randomized controlled trials with rigorous quality assurance protocols. These findings also have implications for the potential implementation of MHRT in LMICs.

## Simple Summary

Cervical cancer remains a significant public health burden, especially in low- and middle-income countries where access to treatment is constrained. Conventional fractionated radiotherapy (CFRT) entails protracted treatment schedules and places considerable demands on both patients and healthcare systems. In contrast, moderately hypofractionated radiotherapy (MHRT) offers a shorter course and has shown promising short- to mid-term efficacy. This approach may improve patient adherence and reduce resource utilization in settings with limited resources. However, given that the current evidence predominantly originates from small or early-phase studies, the long-term efficacy and safety of this approach remain unproven. Consequently, MHRT cannot yet supplant CFRT as the standard of care. Larger, high-quality trials with prolonged follow-up periods, encompassing diverse populations and technologies, are essential.

## 1. Introduction

Cervical cancer (CC) is one of the most common malignancies of the female reproductive system worldwide and continues to be a leading cause of cancer-related mortality, especially in low- and middle-income countries (LMICs), where access to prevention and treatment remains limited, despite efforts to reduce the disease burden in some regions through HPV vaccination and screening programs [1,2,3]. Radiotherapy (RT), encompassing external beam radiotherapy (EBRT) and brachytherapy (BT), plays a critical role in managing CC across all stages of the disease [4,5]. In early-stage disease, definitive RT yields comparable outcomes to surgery [6,7]. For locally advanced cervical cancer (LACC), concurrent chemoradiotherapy (CCRT) remains the standard of care [8]. In metastatic cases, systemic therapy combined with palliative RT is employed to manage symptoms [9]. Therefore, optimizing RT strategies is of significant importance in mitigating the global disease burden of cervical cancer. Conventional fractionated radiotherapy (CFRT) remains the most commonly used approach, typically delivering 45–50.4 Gy in 25 to 28 fractions over 5 to 6 weeks. Although the efficacy of CFRT has been validated through decades of clinical practice, its limitations have become increasingly apparent in modern practice. CFRT’s prolonged 5–6-week course (25–28 fractions) presents dual challenges: for patients, it increases economic and time burdens and the risk of treatment interruptions—particularly in remote areas with limited RT access; for resource-constrained healthcare systems, it strains RT equipment capacity, extends patient wait times, and may exacerbate the risk of tumor progression. Collectively, these challenges represent significant barriers to improving CC care globally [10,11,12].

In recent years, the widespread adoption of precision RT technologies such as intensity-modulated radiotherapy (IMRT) and volumetric modulated arc therapy (VMAT), combined with a deeper understanding of tumor radiobiology, has led to the optimization of RT fractionation regimens as a significant breakthrough in research [13,14]. Among these, hypofractionated radiotherapy (HFRT) has become a central focus in tumor radiation oncology research due to its non-inferior clinical efficacy in certain tumor types and shorter treatment duration [15,16,17]. Based on the single-fraction dose, HFRT can be categorized into two types: (i) moderately hypofractionated radiotherapy (MHRT), which delivers 2.1–4.9 Gy per fraction, and (ii) ultrahypofractionated RT—most commonly exemplified by stereotactic body radiotherapy (SBRT)—characterized by a single-fraction dose ≥ 5 Gy [18]. These modalities differ significantly in their application to CC. Due to its high single-fraction dose and strict requirements for dose gradients in normal tissues, SBRT is currently limited to exploratory salvage treatment for locally recurrent and oligometastatic lesions in CC, resulting in a narrower range of clinical applications [19,20,21]. In contrast, MHRT delivers a more moderate single fraction dose, with improved dose delivery control and enhanced normal tissue tolerance, and its application potential in cervical cancer treatment arguably warrants further exploration.

Accordingly, researchers have attempted to apply MHRT in the management of CC [22]. Current evidence primarily derives from small-sample studies. While its clinical application is expanding and the evidence base continues to grow, key controversies persist. First, regarding MHRT-related toxicities, conflicting results remain: some studies suggest that higher single-fraction doses increase the risk of organ damage, while others indicate that precision RT techniques can effectively mitigate these toxicities [23,24]. A critical gap in this debate is the lack of long-term safety data: most MHRT toxicity studies have follow-up periods of less than five years, and evidence for efficacy and safety beyond this timeframe remains limited. Second, there is no consensus on which CC patient populations are eligible for MHRT, with uncertainty regarding its applicability to all patient groups [24].

Therefore, this article presents a systematic review of research findings associated with MHRT in CC, summarizes the ongoing clinical trials, offers a comprehensive analysis of the principal challenges linked to this therapeutic approach, and provides insights into its future development based on current evidence.

## 2. Methods

### 2.1. Data Sources and Search Strategy

To systematically identify high quality evidence on MHRT for CC, we conducted a structured search of published clinical studies and ongoing or registered trials. Three core databases were searched: PubMed, Google Scholar (first 200 relevant results), and the Web of Science Core Collection. Information on ongoing trials was retrieved from ClinicalTrials.gov. The search strategy used Boolean operators and combined Medical Subject Headings (MeSH) with free text terms to capture relevant disease and intervention synonyms. Core terms included “Uterine Cervical Neoplasms” [MeSH], “Radiotherapy, Hypofractionated” [MeSH], “cervical cancer,” and “moderately hypofractionated radiotherapy.” A representative search string was: (“Uterine Cervical Neoplasms” [MeSH] OR cervical carcinoma) AND (moderately hypofractionated radiotherapy OR moderate hypofractionation radiotherapy). Searches covered each database from inception to 1 October 2025. Studies published after 2020 were prioritized, while seminal earlier trials were retained to ensure continuity of evidence.

### 2.2. Eligibility Criteria

#### 2.2.1. Inclusion Criteria

Literature must meet all the following criteria simultaneously:

- Enrolled patients with CC;
- The study explicitly adopted an MHRT regimen (per-fraction radiation dose: 2.1–4.9 Gy), with comprehensive reporting of dosimetric parameters, treatment-related toxicities, and efficacy endpoint data;
- The literature type was restricted to original research published in peer-reviewed journals or clinical trials registered on ClinicalTrials.gov. Completed registered trials with unreported endpoint data were also included to facilitate the analysis of study design characteristics and research field development trends;
- The literature was published in English.

#### 2.2.2. Exclusion Criteria

Literature was excluded if it met any of the following criteria:

- Unextractable data: Outcome data pertaining to CC patients undergoing MHRT could not be separately extracted from other study cohorts;
- Lack of clinical outcomes: Basic studies focusing exclusively on dosimetry, RT planning, or physics-based simulations, which failed to provide patient follow-up data or clinical endpoint indicators (excluding ongoing trials registered on ClinicalTrials.gov);
- Duplicate publications: Among duplicate publications derived from the same patient cohort, the one with the largest sample size, longest follow-up duration, and most comprehensive outcome indicators was prioritized;
- Non-core evidence types: Single-case reports (*n* = 1), narrative reviews, systematic reviews, expert consensuses, methodological papers, conference abstracts, animal studies, in vitro cell studies, and pure modeling studies. These types of literature were only used as background references and excluded from the core evidence synthesis.

### 2.3. Study Selection and Data Extraction

Study selection followed PRISMA 2020 guidelines, with two independent reviewers screening and cross validating records. Titles and abstracts were screened first, and potentially eligible studies underwent full text review. Disagreements were resolved by consultation with a senior radiation oncologist. The initial search yielded 43 records, including 33 journal articles and 10 ClinicalTrials.gov trials. After eligibility assessment, 15 records were included, comprising 5 published studies and 10 registered trials. Data from published studies were extracted using a validated standardized form, including study design, sample size, baseline characteristics, MHRT parameters, concurrent or adjuvant therapy, follow up duration, toxicity outcomes, and efficacy outcomes. For registered trials, we extracted intervention details, prespecified endpoints, planned sample size, and anticipated completion dates.

### 2.4. Data Synthesis and Statistical Methods

Heterogeneity assessment indicated substantial clinical and methodological variability across studies, precluding the assumptions required for meta analysis. Therefore, we did not perform quantitative pooling and instead conducted a qualitative synthesis of study characteristics and key findings.

## 3. Clinical Application of MHRT in CC

In recent years, MHRT has increasingly garnered significant clinical interest as a therapeutic strategy for CC. Research teams worldwide have conducted many clinical trials to evaluate the safety and efficacy of various MHRT regimens, which differ in fraction dose, total dose, treatment modality, and the inclusion of CCRT. Despite these variations, all trials primarily aim to explore the clinical feasibility of MHRT in the management of CC. This section synthesizes the existing clinical evidence on MHRT for CC, focusing on key regimen-specific differences in efficacy, toxicity profiles, and clinical applicability. The characteristics and outcomes of these studies are summarized in Table 1.

### 3.1. The Existing Evidence: Efficacy and Safety of MHRT in CC

#### 3.1.1. MHRT in Definitive RT for CC

The Indian team led by Mallum A. et al. conducted a prospective trial at Inkosi Albert Luthuli Central Hospital, South Africa (March 2022–March 2023), to compare MHRT with CFRT in patients with LACC (FIGO stage IB3–IVA) in a resource-limited setting [25]. A total of 107 patients were randomized to one of two treatment arms: MHRT (42.72 Gy in 16 fractions; *n* = 53) administered without concurrent cisplatin to minimize treatment-related toxicity, or CFRT (50.5 Gy in 25 fractions; *n* = 54) delivered with concurrent weekly cisplatin (40 mg/m^2^). Both groups subsequently underwent high-dose-rate brachytherapy (HDR-BT) (18 Gy/2f or 21 Gy/3f). MHRT significantly reduced the median treatment duration compared with CFRT (35 vs. 62 days, *p* < 0.001). CFRT was associated with higher rates of grade II gastrointestinal (GI) toxicity (45.8% vs. 33.6%, *p* < 0.001), genitourinary (GU) toxicity (43.9% vs. 36.5%, *p* = 0.01), vaginal stenosis (26.2% vs. 21.5%), and radiation-induced proctitis (21.5% vs. 9.3%, *p* = 0.02). No grade IV toxicities were reported. Clinical outcomes were comparable between the two groups, including 6-month complete clinical response (CCR) (35.8% vs. 32.9%), 12-month recurrence-free survival (RFS) (38.3% vs. 42.1%), and residual disease rates (13.1% vs. 10.3%). These findings indicate that MHRT offers efficacy equivalent to CFRT while reducing treatment duration and toxicity, supporting its feasibility as a cost-effective treatment strategy for CC in resource-limited settings in Sub-Saharan Africa.

In addition to studies conducted in India under resource-limited conditions, the HYPOCx-iRex Trial, carried out by the Division of Radiation Oncology, Department of Radiology, Faculty of Medicine, Siriraj Hospital, Mahidol University, Bangkok, Thailand, is a Phase II, open-label, randomized controlled trial designed to evaluate the safety and efficacy of MHRT (HYPO arm: 44 Gy in 20 fractions) versus CFRT (CVRT arm: 45 Gy in 25 fractions) in patients with LACC. Both arms were administered concurrent weekly cisplatin (40 mg/m^2^ for 5 cycles) plus image-guided adaptive brachytherapy (IGABT), though the specific IGABT fractionation regimen has not been reported in the study [26]. Initially, patients with ≥3 positive pelvic lymph nodes or pathologic lymph nodes at and/or above the common iliac region were excluded; however, acceptable toxicity in the interim analysis allowed an amendment to extend the exclusion limit of metastatic disease to beyond the L2/3 intervertebral disk level. A total of 40 patients were enrolled, with 21 allocated to the MHRT arm and 19 to the CFRT arm. The median follow-up period was 19 months. The overall treatment time (OTT) was significantly shorter in the MHRT arm than in the CFRT arm (39 vs. 47 days, *p* < 0.001). Regarding acute and 18-month late GI toxicities (grade ≥2/≥3), the MHRT arm showed a trend toward higher rates than the CFRT arm (acute: 43%/29% vs. 32%/11%, *p* = 0.53/0.24; late: 26.2%/21.2% vs. 20.6%/14.4%, *p* = 0.537/0.438), although the differences were not statistically significant. No grade ≥ 3 GU toxicity was observed in either arm. Quality of life (QOL) scores were lower in the MHRT arm during treatment but returned to baseline within 3 months after RT. Additionally, the MHRT arm showed a trend toward superior locoregional control, with a significantly higher 24-month para-aortic control rate compared with the CFRT arm (100% vs. 71.2%, *p* = 0.003), while no significant differences in local control or overall survival (OS) were observed between the two groups. In conclusion, MHRT appears feasible for treating LACC. MHRT significantly reduces OTT, exhibits acceptable toxicity, and provides promising locoregional control, although the observed trends in toxicity require further validation.

Although promising results from prospective studies have highlighted the potential of MHRT for CC, not all applications have met expectations. A phase II randomized controlled trial (NCT04831437), led by the Iranian team under Dr. Afsane Maddah Safaei, provides important insights into the risks associated with MHRT. The trial enrolled 59 patients with International Federation of Gynecology and Obstetrics (FIGO) 2018 stage IIA–IIIC1 CC (stage IIIC1 patients with more than three MRI-determined lymphadenopathies and/or with at least one lymph node with a short-axis diameter of 3 cm and/or lymphadenopathy located in the common iliac chains were excluded.), comparing MHRT (40 Gy/15 fractions) with CFRT (45 Gy/25 fractions) [23]. The MHRT arm (*n* = 29) received 3D-conformal radiotherapy (3D-CRT) with the MHRT regimen, concurrently with three weekly cycles of cisplatin (40 mg/m^2^); stage IIIC1 patients additionally received an 8 Gy boost in 3 fractions to gross lymphadenopathies. The CFRT arm (*n* = 30) received 3D-CRT with the standard CFRT dose, up to five weekly cycles of cisplatin (40 mg/m^2^), and a 9 Gy boost in 5 fractions to positive lymph node in stage IIIC1 disease. Both groups subsequently received intravaginal brachytherapy (28 Gy in 4 weekly fractions) beginning one week after chemoradiotherapy, with the MHRT arm showing a significantly shorter overall treatment time (59.6 ± 14.5 days vs. 74.8 ± 10.9 days, *p* = 0.0001). Interim results revealed no significant difference in the primary endpoint—3-month CCR—between the MHRT and CFRT arms: 65.5% (19/29) vs. 66.7% (20/30), respectively (absolute difference 0.011; 95% CI, −0.23 to 0.25; *p* = 0.13), thereby failing to meet the predefined 15% non-inferiority margin. An unplanned subgroup analysis suggested that tumor size influenced MHRT efficacy: patients with a maximum tumor dimension (Dmax) ≤ 5 cm tended to achieve higher CCR rates with MHRT, whereas those with Dmax > 5 cm achieved better outcomes with CFRT (interaction *p* = 0.02). Regarding safety, the MHRT arm exhibited a significantly higher incidence of acute grade ≥ 3 GI toxicity (27.6% vs. 6.7%; *p* = 0.032), primarily severe diarrhea, whereas no significant differences were observed in grade ≥ 3 GU, hematologic, skin toxicities, or acute kidney injury between the two arms. The increased GI toxicity in the MHRT arm was attributed to two factors: (1) both arms employed three-dimensional conformal radiation therapy (3D-CRT), resulting in a bowel volume receiving 45 Gy (V45Gy) that was about twice the QUANTEC-recommended 195 cc; and (2) the higher per-fraction dose in the MHRT arm (~2.67 Gy vs. 1.8 Gy in CFRT). Evidence from multiple clinical studies confirms that intensity-modulated radiation therapy (IMRT) affords superior sparing of organs at risk compared with 3D-CRT. The trial is ongoing with protocol modifications, including the replacement of 3D-CRT with IMRT to improve organ-at-risk sparing and limiting enrollment to patients with Dmax ≤ 5 cm, aiming to further evaluate the non-inferiority and safety of MHRT.

#### 3.1.2. Postoperative Adjuvant RT for CC

Beyond its role in definitive RT, MHRT has also been explored as a postoperative adjuvant RT option in CC. A Korean research team conducted the POHIM-RT trial, a phase II non-randomized multicenter study (NCT03239626), aimed at evaluating the safety and efficacy of MHRT (40 Gy/16 fractions) in postoperative CC patients [27]. This study excluded chemotherapy to minimize its potential interference with toxicity responses. The trial results showed that only 1 patient (1.6%) developed acute ≥grade 3 toxicity (sigmoid colon perforation), which was likely attributed to pre-existing mild intestinal damage rather than high-dose RT. During a median follow-up of 39.5 months, no late toxicities of grade ≥ 3 were observed, and the 3-year DFS rate was 87.1%. However, due to its non-randomized design and small sample size, further phase III trials are required to validate long-term outcomes.

The institution also designed the POHIM-CCRT study (NCT03239613), which focuses on high-risk postoperative patients requiring CCRT (Brachytherapy boost was permitted in the protocol, but extended field radiation, including para-aortic areas, was not allowed). The intervention group received MHRT (40 Gy/16 fractions) plus chemotherapy (9 patients optionally received BT boost with 10–15 Gy in 2–3 fractions) [28]. The median follow-up time was 43 months, and the results revealed that only 2 cases (2.5%) of grade 3 or higher acute adverse events occurred, primarily GI and hematological toxicities. The study also reported that the 3-year DFS rate was 79.3%, and the OS rate was 98.0%. The findings of this multicenter Phase II single-arm trial indicate that postoperative MHRT, in combination with concurrent chemotherapy, is safe and well-tolerated in women with CC. Additional studies are needed to evaluate long-term toxicities and oncological outcomes with prolonged follow-up.

## 4. Ongoing Clinical Trials: Trends and Research Frontiers

Although MHRT has exhibited preliminary potential in the treatment of CC, future research should further refine treatment protocols, assess long-term outcomes, and ensure that MHRT is tailored to diverse patient populations and global healthcare settings. This iterative optimization remains critical to maximizing MHRT’s clinical utility and expanding its global accessibility. Below is an overview of global ongoing clinical trials investigating MHRT in CC, including those completed (i.e., study period concluded) but with unpublished findings. Details of these trials are summarized in Table 2.

### 4.1. Exploration of MHRT Utilizing Adaptive Radiotherapy in Definitive RT for CC

Adaptive Radiotherapy (ART) has been increasingly utilized in oncology owing to its merits of precise localization and real-time adaptation, offering critical technical support for optimizing treatment strategies in CC. Building on previous relevant case reports [22], Peking Union Medical College Hospital, China, has launched the MHARTCC clinical trial (NCT05994300), with the primary goal of developing a personalized MHRT regimen for CC through the utilization of advanced ART technology [29]. The trial will evaluate both efficacy and treatment-related toxicities in patients with cervical squamous cell carcinoma at FIGO stages IB1–IB3, IIA–IIB, or selected stage IIIC1 cases (with a maximum lymph node diameter < 1.5 cm, fewer than three pathological nodes, and no involvement of the common iliac nodes). The treatment regimen comprises MHRT (43.35 Gy in 17 fractions) combined with concurrent weekly cisplatin chemotherapy (40 mg/m^2^) and sequential HDR-BT (dose-fractionation unspecified). The primary endpoint of the trial is acute toxicity, evaluated within three months post-treatment according to CTCAE 5.0. Secondary endpoints include late toxicity at three years, treatment response at one month post-treatment using RECIST 1.1, three-year DFS, OS, and QOL, assessed by the EORTC QLQ-C30 and QLQ-CX24 at the three-year follow-up. Additionally, tumor regression will be quantitatively evaluated by MRI at three months post-RT.

Another ongoing study, the SWIFT-1 trial (NCT06641635), is also being conducted by the same institution. This trial randomizes 440 patients with CC (FIGO stages IB-IIIB; IIIC1 with LNM ≤ 2 cm and no common iliac node involvement) to receive either MHRT (43.35 Gy in 17 fractions with ART) or CFRT (45 Gy in 25 fractions), both combined with weekly cisplatin chemotherapy (40 mg/m^2^) (±PD-1 inhibitors) and HDR-BT (dose-fractionation unspecified) [30]. The primary endpoint is 3-year progression-free survival (PFS). Secondary endpoints include 3-month complete response rate (CRR), acute toxicity, 3-month treatment cost, as well as 3-year late toxicity, OS, locoregional progression-free survival (LR-PFS), metastasis-free survival, cancer-specific survival (CSS), and QOL. This trial uniquely validates the “precision + efficiency” of ART-guided MHRT in LACC, emphasizing the reduction in treatment duration rather than solely addressing target motion, and aims to provide Class I evidence for non-inferior oncologic outcomes with shorter treatment regimens.

### 4.2. Investigation of MHRT in Special Sub-Groups of CC Patients

Researchers are now seeking to expand the indications for MHRT. Its applications have evolved from a traditional focus on low-risk CC patients to encompass several novel clinical scenarios. Below, we analyze each scenario in detail.

#### 4.2.1. Exploration of MHRT in Postoperative High-Risk CC

High-risk postoperative CC patients typically require adjuvant chemoradiotherapy. Building on the favorable results from the POHIM-CCRT and RT trials, a Korean research team designed the POHIM-P3 phase III randomized controlled trial (NCT06509724) [31]. The study plans to randomize 248 patients (1:1 allocation): the control group will receive CFRT (45–50.4 Gy in 25–28 fractions) with 5–6 cycles of cisplatin chemotherapy, while the experimental group will receive MHRT (40 Gy/16 fractions) with 3 cycles of cisplatin chemotherapy. BT is allowed in both groups (dose-fractionation unspecified). The primary endpoint is 3-year LCR, with secondary endpoints including 3-month acute toxicity, from 3 months post-treatment up to 5-year chronic toxicity, 5-year OS, DFS and QOL. The trial is ongoing, and only the protocol details have been published to date.

#### 4.2.2. Refractory/Bulky CC Patients

For patients with treatment-naive bulky or limited metastatic CC, treatment options remain suboptimal. The MCC-23-GYN-10 trial (NCT06331468), a Phase II clinical trial initiated by the University of Kentucky and led by Dr. Denise Fabian, aims to evaluate the feasibility, efficacy, and safety of MHRT in 20 treatment-naive female patients with CC—specifically FIGO 2018 Stage IB3, II, or IIIA-IIIC1 bulky tumors (≥6 cm) or Stage IVB disease with limited metastatic burden (not requiring urgent systemic therapy) [32]. Its core regimen is a MHRT scheme: 8 fractions of pelvic IMRT (4.56 Gy/fraction, Monday–Thursday for 2 weeks); 2 cycles of cisplatin (40 mg/m^2^, maximum 70 mg per dose, Day 1/Day 8 ± 1, with dose reduction to 30 mg/m^2^ permitted for toxicity); and 4 fractions of HDR-BT (7 Gy/fraction, twice weekly with ≥72 h intervals, 2 weeks total), with the entire radiation course ≤ 30 ± 2 days. This design cuts standard EBRT from 25 fractions to 8 and cisplatin from 5–6 cycles to 2, aiming to improve treatment adherence. Primary endpoint: MRI-assessed CRR per RECIST v1.1 at Day 60 (1 month post-treatment). Secondary endpoints include 3-month MRI-assessed CR rate, 2-year PFS, OS, and treatment tolerability. Exploratory endpoint: change in circulating tumor cell (CTC) levels pre-treatment, 1-month, and 3-month post-treatment. By including patients with bulky (≥6 cm) or limited metastatic CC, this trial aims to expand treatment options for this difficult-to-treat population. If successful, it could provide a new treatment option for such patients, addressing a significant evidence gap for the application of MHRT in this population. The trial is expected to conclude by July 2028.

#### 4.2.3. Chemotherapy-Intolerant CC Patients

A phase I/II study (NCT05210270) conducted by University of Santo Tomas Hospital (Philippines) aims to address the gap in chemotherapy-free MHRT for patients with chemotherapy-intolerant LACC (2018 FIGO Stage IIIA-IIIC1, IVA; excluding IIIC2 and distant metastasis) [33]. The treatment regimen includes pelvic MHRT (40 Gy in 15 fractions via IMRT), SIB with the dose stratified by lymph nodes location (48 Gy for common iliac/inguinal nodes, 45 Gy for internal/external iliac nodes) to compensate for efficacy loss from omitted chemotherapy, and sequential HDR-BT (6.5–7.5 Gy × 4 fractions). For study design, Phase I adopts a 3 + 3 dose-escalation design targeting the maximum tolerated dose (MTD) of lymph node SIB (defined as ≤33% dose-limiting toxicity within 4 months) as the primary endpoint; Phase II uses a Simon two-stage design (planned 55 patients, accounting for 10% attrition) with 3-month CRR (target ≥ 84%) as the primary endpoint. Secondary endpoints include 4-month and 5-year toxicity, 5-year PFS, LPFS, metastasis-free survival (MFS), CSS, OS, and QOL. This trial provides a chemotherapy-free treatment option for LACC patients with poor organ function or chemotherapy contraindications, with an estimated completion in March 2030.

#### 4.2.4. Evaluation of MHRT for CC Patients Across Diverse Resource Settings

The global disparity in CC treatment is evident, with high-income countries (HICs) prioritizing efficiency and resource optimization within their healthcare systems. In contrast, LMICs face significant challenges, including inadequate RT infrastructure and long patient travel distances, leading to high treatment abandonment rates. Current research focuses on adapting MHRT to these diverse resource settings, with ongoing trials evaluating its feasibility and effectiveness.

##### MHRT in HICs: Feasibility and Safety in Public Healthcare Systems

The Phase 1/2 interventional clinical trial (NCT06529809), initiated by Washington University School of Medicine and supported by the National Cancer Institute (NCI), is a feasibility study aimed at enhancing local control and survival outcomes in patients with locally advanced CC by reducing the overall treatment duration. The trial enrolls patients with FIGO 2018 stages IB3–IVA [34]. The intervention involves Accelerated Brachytherapy combined with Hypofractionated Radiotherapy and Concurrent Chemotherapy (ABC-RT), which combines hypofractionated EBRT with concurrent chemotherapy and early upfront, image-guided BT—a novel strategy that reverses the conventional sequence of “EBRT followed by BT.” This approach shortens the overall treatment course while maintaining therapeutic dose intensity. The regimen begins with 2 fractions of HDR-BT prior to EBRT-based chemoradiotherapy, with a total of 6 HDR-BT fractions (7.3 Gy per fraction). The specific MHRT schedule is as follows: 19.05 Gy in 15 fractions for the central pelvic region, 40 Gy in 15 fractions for nodal basins, and 48 Gy in 15 fractions for grossly LNM. Concurrent chemotherapy follows standard clinical guidelines, and the total treatment duration is compressed to 36–42 days, directly addressing the clinical challenge of prolonged treatment reducing local control. The primary endpoint is the incidence of grade 3 or higher late GI and GU adverse events during the 91-day to 2-year follow-up period. Secondary endpoints include 2-year local recurrence-free survival (LRFS), regional recurrence-free survival (RRFS), and PFS. Beyond evaluating the safety and efficacy of the accelerated regimen, this study also lays the foundation for future research on combining RT with immunotherapy.

Additionally, the Phase II interventional trial HEROICC (NCT04583254), sponsored by the London Health Sciences Centre Research Institute and St. Joseph’s Lawson Research Institute, aims to evaluate the feasibility of MHRT within the Canadian healthcare system [35]. The study will enroll 48 patients with stage IIIC1 cases required to meet all the following criteria: largest node is less than 3 cm; less than 3 pathological nodes; no nodes located in the common iliac chain; and cervical confined or with parametrial invasion. Participants will be randomized into two groups: the experimental group will receive MHRT (40 Gy/15 fractions) with weekly cisplatin (40 mg/m^2^, maximum 5 cycles), followed by HDR-BT (dose-fractionation unspecified); while the control group will undergo CFRT (45 Gy/25 fractions) with the same concurrent cisplatin regimen and HDR-BT (dose-fractionation unspecified). This study focuses on the practical feasibility of the shortened RT regimen in the Canadian context, with a core focus on patient accrual. The primary endpoint is trial feasibility, defined as enrolling and randomizing 48 patients within 3 years. Secondary endpoints include toxicity assessments at both 3 months and 3 years, cancer downstaging after 3 years of EBRT, and long-term outcomes include 8-year PFS, LPFS, MFS, CSS, OS, and QOL. The trial is expected to conclude by December 2028.

##### MHRT in LMICs: Feasibility and Safety in Public Healthcare Systems

A phase II randomized controlled trial (NCT04831437), conducted by Tehran University of Medical Sciences (Iran), aims to shorten the treatment course while verifying non-inferior efficacy and acceptable toxicity for patients with CC [36]. The study will enroll 60 patients with FIGO stage IB–IIIC CC (with IIIC1 limited to ≤3, lymph nodes < 3 cm in diameter and no common iliac LNM, and IIIB limited to hydronephrosis with normal creatinine clearance): the experimental group will receive MHRT with 40 Gy in 15 fractions plus 3 cycles of cisplatin (40 mg/m^2^), while the control group will receive CFRT with 45 Gy in 25 fractions plus 5 cycles of cisplatin (40 mg/m^2^). Both groups will undergo HDR-BT (28 Gy/4 f) one week after the completion of EBRT. The primary endpoints include early toxicity (within 3 months after treatment completion) and early radiological response (at 3 months after treatment completion). The secondary endpoints include late toxicity (at 1 and 3 years after treatment completion), 5-year disease-specific survival (DSS), PFS, and OS. The study is expected to conclude by March 2028. The interim results of this trial did not meet the prespecified criteria to establish the non-inferiority of MHRT compared with CFRT. To address the previously observed increase in acute GI toxicity in the MHRT group and to further validate efficacy, the trial is continuing patient accrual, replacing 3D-CRT with IMRT to evaluate the efficacy and safety of the modified MHRT regimen [23].

Separately, a Phase II interventional trial (NCT04070976) conducted at Mexico’s National Institute of Cancerology (INCan) aims to compare the safety and treatment response of MHRT versus CFRT in the setting of concurrent chemoradiotherapy, with both approaches followed by sequential BT, in patients with clinically staged III CC (FIGO stages IIIA, IIIB, IIIC1) [37]. The primary objectives of this study are to assess the safety and efficacy of CCRT followed by sequential BT in this patient cohort, analyze treatment-related costs, QOL, and survival outcomes, and evaluate the therapeutic efficacy and potential adverse events of MHRT. Eligible patients will be randomly assigned to one of two groups: the intervention arm will receive MHRT (37.5 Gy in 15 fractions) combined with concurrent weekly cisplatin chemotherapy (40 mg/m^2^), followed by sequential BT (28 Gy); the control arm will receive standard fractionated EBRT (50 Gy in 25 fractions) with concurrent weekly cisplatin chemotherapy (40 mg/m^2^), followed by sequential BT (28 Gy). The primary endpoint is the 2-year incidence of acute and late toxicity, while secondary endpoints include 2-year treatment efficacy, DFS, OS, patient satisfaction, and direct and indirect treatment-related costs.

A phase II randomized trial (NCT03750539) conducted in Honduras and Mexico evaluates MHRT regimens for patients with FIGO stage IB2–IIB CC [38]. This trial compares MHRT (37.5 Gy delivered in 15 fractions) with CFRT (45 Gy delivered in 25 fractions); all patients receive concurrent weekly cisplatin (40 mg/m^2^) during RT, followed by type II or type III open radical hysterectomy 4–6 weeks after RT completion. The primary endpoint is 2-year acute and late toxicity. Secondary endpoints include 1-year surgical complications, 2-year OS, 2-year DSS, and treatment equivalence. The trial is expected to be completed by 10 November 2025, and its results will determine whether MHRT achieves efficacy comparable to CFRT in this patient population.

## 5. Discussion

MHRT is being explored as a potential therapeutic strategy for CC, shortening the total treatment course and optimizing healthcare resource utilization. Early evidence from small cohorts with limited follow-up suggests that acute toxicities associated with MHRT are manageable and generally well tolerated in clinical practice. However, major unresolved controversies persist, including uncertainties regarding radiobiological feasibility, scarce data in high-risk patient populations, methodological limitations and evidence gaps, and variability in technical resources. This review systematically summarizes the current clinical application of MHRT in CC, discusses core controversies and future development directions, and aims to provide a reference for evidence-based and context-appropriate clinical practice and exploration.

### 5.1. Radiobiological Feasibility of MHRT in CC

Radiobiological feasibility is central to evaluating MHRT in CC. Dose–response under fractionation is commonly described by the linear–quadratic (LQ) model: S = e^(−αD − βD^2^). The α/β ratio is a key determinant of fractionation sensitivity: lower values favor hypofractionation, while higher values align with CFRT [39,40]. Squamous cell carcinoma, the predominant histotype in CC, was traditionally assigned an α/β ratio of ~10 Gy (less favorable for hypofractionation), but emerging data reveal biological heterogeneity with reported values ranging from 4 to 10 Gy—supporting a rationale for MHRT, though the true α/β distribution and its clinical implications still require further investigation [41].

Quantitative comparison via LQ-derived equivalent dose in 2 Gy fractions (EQD2, EQD2 = D × (d + α/β)/(2 + α/β); D = total dose, d = single-fraction dose) reveals non-trivial EQD2 reductions with MHRT for early-responding tissues. For late-reacting tissues (α/β = 3 Gy), CFRT (45–50.4 Gy in 25–28 fractions) yields an EQD2 of 43.2–48.4 Gy versus 44.0 Gy for MHRT (40 Gy in 16 fractions). For early-reacting tissues (α/β = 10 Gy), CFRT’s EQD2 is 44.3–49.5 Gy versus 41.7 Gy for MHRT, which may translate into a risk of inferior local control, particularly in biologically aggressive disease.

Notably, accelerated tumor repopulation in CC initiates ~19 days (range 11–22 days) post-RT [42]. Clinical data show a median OTT of 59 days, with only 38.2% of patients completing treatment within 56 days [43]; each 1-day OTT prolongation correlates with a 0.8% decrease in 3-year OS and 1.0% reduction in pelvic control [43,44]. By shortening overall treatment time and modestly increasing dose per fraction, MHRT could narrow the window for accelerated repopulation and partly compensate for lower EQD2; however, the magnitude of this trade-off remains uncertain. Future optimization of dose-fractionation parameters should integrate tumor proliferation kinetics and individual biological characteristics.

### 5.2. Limited Evidence in High-Risk Populations

Most exploratory MHRT studies used stringent eligibility criteria. High-risk features (bulky tumors ≥ 5 cm, multiple nodal metastases, or para-aortic/common iliac involvement) were typically excluded, leaving limited real-world evidence and constraining generalizability. Key trial examples illustrate this limitation: the Thai HYPOCx-iRex trial initially excluded patients with ≥3 positive pelvic nodes or common iliac/para-aortic metastases (later amending exclusion to lesions above L2/3) [26]; the Korean POHIM-CCRT study (NCT03239613) prohibited para-aortic extended-field RT [28]; and an Iranian randomized trial excluded stage IIIC1 patients with >3 positive nodes, nodal short-axis ≥ 3 cm, or common iliac involvement [23]. The Iranian study further highlighted MHRT’s limitations in high-risk patients: for tumors > 5 cm, the CR rate was 37.5% in the MHRT group versus 69.2% in the CFRT group (*p* < 0.05), creating a clinical dilemma in identifying beneficiaries versus those at risk of undertreatment/overtreatment. Mechanistically, high-risk features conflict with MHRT’s profile: multiple nodal metastases expand target volumes, increasing normal tissue (small intestine, bone marrow) exposure—exacerbated by MHRT’s higher single-fraction dose—while pelvic/para-aortic involvement requires renal hilum-level fields, elevating dose to critical organs (kidneys, spinal cord). With few studies conducting stratified analyses for high-risk factors, MHRT’s performance across risk strata remains unclear. Until prospective subgroup data are available, MHRT in high-risk disease should be restricted to clinical trials or carefully selected patients with robust image-guided planning and brachytherapy support.

### 5.3. Research Design Limitations and Evidence Gaps

Current MHRT studies in CC suffer from protocol limitations that undermine reliability and clinical translation. Regimen heterogeneity is a core issue: ICBT, integral for local dose escalation, was administered to only 11.4% of patients in the POHIM-CCRT study versus standard use in other trials, invalidating cross-trial efficacy comparisons [28]. Inconsistency in concurrent chemotherapy (cisplatin-based regimens omitted in one MHRT arm but standard in the control arm) further complicates interpreting treatment outcomes [25]. Despite heterogeneity, data support MHRT’s feasibility in selected contexts: in the definitive setting, the South African Mallum et al. study reported 6-month CCR rates of 35.8% (MHRT) vs. 32.9% (CFRT) and 12-month RFS of 38.3% vs. 42.1% (no significant differences), while the Thai HYPOCx-iRex trial (median follow-up 19 months) showed no differences in LCR or OS. In the adjuvant setting, POHIM-RT reported 3-year DFS of 87.1% with MHRT alone, and POHIM-CCRT achieved 3-year DFS of 79.3% and OS of 98.0% with MHRT + concurrent chemotherapy [25,26,27,28].

Small sample sizes and relatively short follow up durations, typically less than three years, remain major limitations of current studies in this area. Importantly, late toxicities after radiation therapy for CC can emerge months to years after treatment, and some very late complications arise even later, often three to five years or more after completion of therapy. In addition, several reports lack long term tumor control data for high risk patients who did not receive ICBT or chemotherapy. This gap reduces the evidentiary strength of the conclusions and limits the extent to which the findings can inform or support guideline recommendations.

### 5.4. Technical Resource Heterogeneity: Challenges in Global Implementation

MHRT’s clinical implementation relies on precise dose delivery, which limits high-dose exposure to organs at risk (OARs), such as the small intestine and bladder. Insufficient technical precision or suboptimal planning drastically increases normal tissue toxicity, as illustrated by the Iranian study: using 3D-CRT, acute grade ≥ 3 GI toxicity was 27.6% in the MHRT group versus 6.7% in the CFRT group (*p* = 0.032), with intestinal V45Gy (195 cc) twice the QUANTEC-recommended limit—exacerbated by MHRT’s higher single-fraction dose [23].

Global resource inequities pose major barriers, particularly in LMICs. While MHRT theoretically alleviates resource burdens by shortening courses, LMICs face substantial technical and quality-assurance barriers: even with basic IMRT equipment, lack of standardized calibration and personnel training prevents effective implementation. Directly transferring HICs protocols to LMICs settings without robust QA infrastructure and workforce training may increase OAR. Successful global implementation of MHRT will require improved technical accessibility and systematic workforce training, not just protocol replication [45,46].

### 5.5. Cutting-Edge Clinical Trials: Addressing Gaps and Emerging Challenges

Ongoing trials are addressing key evidence gaps while exposing new challenges. Targeting high-risk populations, the U.S. MCC-23-GYN-10 trial (NCT06331468) enrolls patients with tumors ≥ 6 cm, and the Philippine NCT05210270 trial explores SIB for chemotherapy-intolerant patients [32,33]. These studies broaden applicability but lack standardized screening criteria; critical questions (dose adjustment thresholds for bulky tumors, safe SIB limits) require multicenter validation. Technological empowerment is another key direction: the Chinese MHARTCC trial (NCT05994300) combines ART with MHRT for stage IB1–IIIC1 CC, dynamically adjusting target volumes to reduce OARs dose and focusing on acute toxicity [29]. The Chinese SWIFT-1 trial (NCT06641635) (*n* = 440) compares 3-year PFS between ART-guided MHRT and CFRT, but its >3-year follow-up delays short-term evidence generation. Notably, ART capacity remains limited in most LMICs, restricting scalability [30].

## 6. Conclusions

In summary, although MHRT confers the important advantages of a shorter treatment course and more efficient resource utilization in the management of CC and has demonstrated feasibility in initial studies, the available evidence remains inadequate to endorse its widespread replacement of conventional fractionation regimens. The majority of existing studies involve patients at relatively low risk, the boundaries of the eligible patient population remain indistinct, and data on long-term disease control and late toxicity remain scarce. Future efforts should comprise large-scale, multicentre, long-term follow-up randomized controlled trials that stratify patients by risk category and across diverse technological settings in order to thoroughly assess the efficacy-toxicity balance of MHRT, thereby defining the appropriate patient cohorts and clinical scenarios and progressively facilitating its wider clinical integration.

## Figures and Tables

**Table 1:** Key Information Summary of Studies of MHRT-Related Studies for CC.

Publication, Year	Trial Details	Population	Treatment Plan	Primary Outcome	
				Efficacy Indicators	Safety Indicators
Mallum, A et al., 2025 [25]	RCT; *n* = 107; follow-up 12 months	FIGO IB3-IVA	Intervention arm (*n* = 53): VMAT 42.72 Gy/16 f + selective dose boost 10 Gy/5 f + HDR-BT 18 Gy/2f or 21 Gy/3f + no concurrent weekly cisplatin Control arm (*n* = 54): VMAT 50.50 Gy/25 f + selective dose boost 10 Gy/5 f + HDR-BT 18 Gy or 21 Gy + Concurrent weekly cisplatin (40 mg/m^2^)	6-month CCR: Intervention arm 35.8% vs. Control Arm 32.9%; 6-month partial clinical response: Intervention arm 12.2% vs. Control arm 11.2%; 6-month residual disease: Intervention arm 0.9% vs. Control arm 0%; 12-month residual disease: Intervention arm 13.1% vs. Control arm 10.3%; 12-month RFS rate: Intervention arm 38.3% vs. Control arm 42.1% (*p* = 0.540); 12-month recurrence rate: Intervention arm 10.3% vs. Control arm 8.4%; 12-month mortality: Intervention arm 0.9% vs. Control arm 0%.	Grade 2 GI toxicity rate: Intervention arm 33.6% vs. Control arm 45.8% (*p* < 0.001) Radiation proctitis rate: Intervention arm 9.3% vs. Control arm 21.5% (*p* = 0.02) Vaginal stenosis rate: Intervention arm 21.5% vs. Control arm 26.2% (no significant *p*-value reported) Grade 2 GU toxicity rate: Intervention arm 36.5% vs. Control arm 43.9% (*p* = 0.01) Grade 3 skin toxicity rate: Intervention arm 7.5% vs. Control arm 12.1% (*p* = 0.012)
Dankulchai P et al., 2025 [26]	Phase II RCT; interim analysis; *n* = 40; median follow-up 19 months	LACC(Initially excluded: ≥3 positive pelvic lymph nodes or pathologic lymph nodes at/above the common iliac region; metastatic-disease exclusion later extended beyond the L2/3 disk.)	Intervention arm (*n* = 21): IMRT/VMAT 44 Gy/20 f + SIB for pelvic and paraaortic LNM 53/55.4 Gy (2.65/2.77 Gy/fraction) + concurrent weekly cisplatin (40 mg/m^2^ × 5 cycles) + IGABT (fractionation unspecified) Control arm (*n* = 19): EBRT 45 Gy/25 f + SIB for pelvic and paraaortic LNM 55/57.5 Gy (2.2/2.3 Gy/fraction) + concurrent weekly cisplatin (40 mg/m^2^ × 5 cycles) + IGABT (fractionation unspecified)	3-month tumor response: Intervention arm vs. Control arm (*p* > 0.05); 24-month para-aortic control: Intervention arm 100% vs. Control arm 71.2% (*p* = 0.003); 24-month local control, pelvic control, lymph nodes control, locoregional control, distant metastatic control, event-free survival, and OS: Intervention arm vs. Control arm (*p* > 0.05);	Acute toxicity: GI (G2+) Intervention arm: 43% vs. Control arm: 32%, *p* = 0.53; GI (G3+) Intervention arm: 29% vs. Control arm: 11%, *p* = 0.24; Hematologic (G3+) Intervention arm: 24% vs. Control arm: 11%, *p* = 0.41; Late toxicity: 18-month actuarial GI (G2+) Intervention arm: 26.2% vs. Control arm: 20.6%, *p* = 0.537; 18-month actuarial GI (G3+) Intervention arm: 21.2% vs. Control arm: 14.4%, *p* = 0.438;
Maddah Safaei A et al., 2024 [23]	Phase II RCT; *n* = 59; interim analysis	FIGO 2018 IIA-IIIC1; (excluded: patients with >3 MRI-detected lymph nodes, or any lymph nodes ≥ 3 cm short-axis, or nodes in the common-iliac chains)	Intervention arm (*n* = 29): 3D-CRT 40 Gy/15 f + LNM boost 8 Gy/3 f + concurrent weekly cisplatin 40 mg/m^2^ (3 cycles) + ICBT 28 Gy/4 f Control arm (*n* = 30): 3D-CRT 45 Gy/25 f + LNM boost 9 Gy/5 f + Concurrent weekly cisplatin 40 mg/m^2^ (up to 5 cycles) + ICBT 28 Gy/4 f	3-month CCR: Intervention arm 65.5% vs. Control arm 66.7% (*p* = 0.13).	Acute toxicity: Intervention arm: G3+ 44.8% vs. Control arm: 30%, *p* = 0.24; Intervention arm: GI G3+ 27.6% vs. Control arm: 6.7%, *p* = 0.032 Mortality: 3 cases (Intervention arm: 1 case of fatal arrhythmia secondary to diarrhea-induced hypokalemia; Control arm: 2 cases, 1 from sepsis due to ascites and peritonitis, 1 from acute myocardial infarction)
Cho WK et al., 2025 [27]	Multicenter Phase II single-arm trial; *n* = 61; median follow-up 39.5 months	Postoperative CC with high-risk factors;	IMRT 40 Gy/16 f + Selective HDR ICBT boost (10–15 Gy/2–3 f)	3-year DFS: 87.1%; Disease-related mortality: None reported; Recurrence rate: 9.8% (3 regional recurrences, 3 lung metastases).	Acute toxicity: G3+ 1.6% (1 case of G4 sigmoid perforation); G2+ GI 80.3%, GU 39.3%, hematologic 9.8%; Late toxicity: No G3+; G2+ GI/GU both 34.4%, G1 lymphedema 6.6%
Cho WK, et al., 2024 [28]	Multicenter Phase II single-arm trial; *n* = 79; median follow-up 43 months	Postoperative CC with high-risk factors; (extended field radiation including para-aortic areas prohibited)	IMRT 40 Gy/16 f + concurrent chemotherapy (weekly cisplatin 40 mg/m^2^ × 3 cycles or fluorouracil 1000 mg/m^2^ days 1–5 + cisplatin 60 mg/m^2^ × 2 cycles) + Selective BT boost (10–15 Gy/2–3 f)	3-year DFS: 79.3%, 3-year OS: 98%; Recurrence rate: 20.3% (distant metastasis: 12.7%).	Acute toxicity: G3+ 2.5% (1 case G3 GI + anemia, 1 case G3 anemia); G2+ GI 76%, GU 19% (all G1), hematologic 29.1% Late toxicity: G2+ GI 31.6%, GU 24.1%, lymphedema 17.8%

Abbreviation Notes: ① RCT: Randomized Controlled Trial; ② FIGO: International Federation of Gynecology and Obstetrics; ③ VMAT: Volumetric Modulated Arc Therapy; ④ HDR-BT: High-Dose Rate Brachytherapy; ⑤ CCR: Complete Clinical Response; ⑥ RFS: Recurrence-Free Survival; ⑦ GI: Gastrointestinal; ⑧ GU: Genitourinary; ⑨ LACC: Locally Advanced Cervical Cancer; ⑩ IMRT: Intensity-Modulated Radiotherapy; ⑪ SIB: Simultaneous Integrated Boost; ⑫ LNM: Lymph Node Metastasis; ⑬ IGABT: Image-Guided Adaptive Brachytherapy; ⑭ EBRT: External Beam Radiation Therapy; ⑮ OS: Overall Survival; ⑯ G2+: Grade 2 and above; ⑰ G3+: Grade 3 and above; ⑱ 3D-CRT: 3-Dimensional Conformal Radiation Therapy; ⑲ ICBT: Intracavitary Brachytherapy; ⑳ CC: Cervical Cancer; ㉑ HDR ICBT: High-Dose Rate Intracavitary Brachytherapy; ㉒ DFS: Disease-Free Survival; ㉓ G4: Grade 4; ㉔ BT: Brachytherapy; ㉕ G3: Grade 3; ㉖ G1: Grade 1.

**Table 2:** Summary of Global Ongoing Clinical Trials on MHRT for CC.

Identifier	Institution	Trial Type	Population	Intervention Regimen	Endpoint		Estimated Completion Date
					Primary Endpoint	Secondary Endpoints	
NCT05994300 [29]	Peking Union Medical College Hospital	Phase 1; Single arm	FIGO stage IB1, IB2, IB3, IIA, IIB or IIIC1 (with a maximum lymph node diameter < 1.5 cm, fewer than three pathological nodes, and no involvement of the common iliac nodes); planned enrollment of 30 patients	Single arm: MHRT-ART (43.35 Gy/17 f) + Concurrent weekly cisplatin (40 mg/m^2^) + HDR-BT (dose-fractionation unspecified)	3-month acute toxicity	1-month response evaluation 3-month tumor regression 3-year late toxicity, DFS, OS, and QOL.	31 December 2025
NCT06641635 [30]	Peking Union Medical College Hospital	Phase 3; RCT	FIGO stage IB-IIIB, or IIIC1 (LNM ≤ 2 cm, without common iliac LNM); planned enrollment of 440 patients	Intervention arm: MHRT-ART (43.35 Gy/17 f) + Concurrent weekly cisplatin (40 mg/m^2^) or PD-1 inhibitors + HDR-BT (dose-fractionation unspecified) Control arm:EBRT (45 Gy/25 f) + Concurrent weekly cisplatin (40 mg/m^2^) or PD-1 inhibitors + HDR-BT (dose-fractionation unspecified);	3-year PFS	3-month acute toxicity, treatment cost, CRR 3-year late toxicity, OS, LR-PFS, MFS, CSS, and QOL.	31 October 2029
NCT06509724 [31]	Samsung Medical Center	Phase 3; RCT	Radical hysterectomy + pelvic lymphadenectomy, and meet pelvic LNM, parametrial involvement, or positive surgical margins; planned enrollment of 248 patients	Intervention arm: EBRT (40 Gy/16 f) + Concurrent weekly chemotherapy (3 sessions, dose unspecified) ± BT (dose-fractionation unspecified) Control arm: EBRT (40–50.4 Gy/25–28 f) + Concurrent weekly chemotherapy (5–6 sessions, dose unspecified) ± BT (dose-fractionation unspecified)	3-year LCR	3-month and 5-year toxicity; 5-year OS, DFS and QOL.	31 December 2032
NCT06331468 [32]	University of Kentucky	Phase 2; Single arm	FIGO stage IB3, II, or IIIA-IIIC1 bulky (≥6 cm) or Stage IVB; planned enrollment of 20 patients	Single arm: IMRT (36.5 Gy/8 f, once daily Mon-Thu, 4 fractions/week for 2 weeks) + Concurrent cisplatin (2 IV infusions on Day 1 and Day 8 ± 1 day, starting dose 40 mg/m^2^, dose reduction to 30 mg/m^2^ allowed as needed) + HDR-BT (28 Gy/4f, 2x weekly on weekdays, ≥72 h interval between sessions)	CRR at 1 month post-treatment	3-month CRR; 2-year PFS, OS and toxicity.	1 July 2028
NCT05210270 [33]	University of Santo Tomas Hospital	Phase 1/2; Single arm	FIGO stage IIIA-IIIC1 or IVA; planned enrollment of 55 patients	Single arm: Phase 1 (Dose Escalation Study): EBRT (40 Gy/15 f) + nodal SIB (45 Gy [first dose level], 48 Gy [second dose level]) + HDR-BT (26–30 Gy/4 f); Phase 2 (Efficacy Study): EBRT (40 Gy/15 f) ± nodal SIB (45–48 Gy, per Phase 1 results) + HDR-BT (26–30 Gy/4 f)	Phase1: MTD (4-month dose-limiting toxicity <33%); Phase2: 3-month CRR	4-month and 5-year toxicity; 5-year PFS, LPFS, MFS, CSS, OS and QOL.	March 2030
NCT06529809 [34]	Washington University School of Medicine	Phase 1/2; Single arm	FIGO stage IB3-IVA; planned enrollment of 50 patients	Single arm: EBRT (central pelvis 19.05 Gy/15f, nodal basins 40 Gy/15f, grossly positive lymph node 48 Gy/15f) + HDR-BT (7.3 Gy × 6f); Concurrent chemotherapy follows standard of care (not protocol-mandated)	91-day to 2-year acute and late toxicity	2-year LRFS, RRFS and PFS.	31 December 2031
NCT04583254 [35]	London Health Sciences Centre/Lawson Institute	Phase 2 RCT	FIGO stage IB2, IB3, IIA, IIB or IIIC1 (IIIC1 must meet all criteria below: largest node < 3 cm; pathological nodes < 3; no common iliac chain nodes; tumor confined to cervix or with parametrial invasion.); planned enrollment of 48 patients	Intervention arm: EBRT (40 Gy/15 f) + Concurrent weekly cisplatin (40 mg/m^2^, a maximum of 5 cycles) + HDR-BT (dose-fractionation unspecified) Control arm: EBRT(45 Gy/25 f) + Concurrent weekly cisplatin (40 mg/m^2^, a maximum of 5 cycles) + HDR-BT (dose-fractionation unspecified)	Feasibility	3-month and 3-year toxicity; 3.5-year tumor response; 3-year cancer downstaging throughout; 8-year LPFS, MFS, CSS, PFS, OS, and QOL.	14 December 2028
NCT04831437 [36]	Tehran University of Medical Sciences	Phase 2 RCT	FIGO stage IB, IIA, IIB, IIIA, IIIB, IIIC1 (if less than 3 lymph nodes with size less than 3 cm, and without involvement of common iliac chain); planned enrollment of 60 patients	Intervention arm: EBRT (40 Gy/15 f) + Concurrent weekly cisplatin (40 mg/m^2^, 3 cycles) + HDR-BT (28 Gy/4 f) Control arm: EBRT (45 Gy/25 f) + Concurrent weekly cisplatin (40 mg/m^2^, 5 cycles) + HDR-BT (28 Gy/4 f)	3-month: acute toxicity, imaging-based tumor response	1-year and 3-year Late toxicity; 5-year PFS, DSS and OS.	March 2028
NCT04070976 [37]	National Institute of Cancerología (Mexico)	Phase 2 RCT	FIGO stage IIIA, IIIB or IIIC1; planned enrollment of 82 patients	Intervention arm: EBRT (37.5 Gy/15 f) + Concurrent weekly cisplatin (40 mg/m^2^) + BT (28 Gy) Control arm: EBRT (50 Gy/25 f) + Concurrent weekly cisplatin (40 mg/m^2^) + BT (28 Gy)	2-year acute and late toxicity	2-year treatment efficacy, DFS, OS, Satisfaction assessed by EORTC, and costs.	30 December 2024
NCT03750539 [38]	National Institute of Cancerología (Mexico)	Phase 2 RCT	FIGO stage IB2-IIB; planned enrollment of 100 patients	Intervention arm: EBRT (37.5 Gy/15 f) + Concurrent weekly cisplatin (40 mg/m^2^) + radical hysterectomy Control arm: EBRT (45 Gy/25 f) + Concurrent weekly cisplatin (40 mg/m^2^) + radical hysterectomy	2-year acute and late toxicity	1-year surgical complications; 2-yearOS, DSS, and equivalent treatment.	10 November 2025

Abbreviation Notes: ① MHRT-ART: Moderate Hypofractionated Radiation Therapy-Adaptive Radiation Therapy; ② HDR-BT: High-Dose Rate Brachytherapy; ③ DFS: Disease-Free Survival; ④ OS: Overall Survival; ⑤ QOL: Quality of Life; ⑥ RCT: Randomized Controlled Trial; ⑦ LNM: Lymph Node Metastasis; ⑧ PD-1: Programmed Death-1; ⑨ EBRT: External Beam Radiation Therapy; ⑩ PFS: Progression-Free Survival; ⑪ CRR: Complete Response Rate; ⑫ LPFS: Locoregional Progression-Free Survival; ⑬ MFS: Metastasis-Free Survival; ⑭ CSS: Cancer-Specific Survival; ⑮ BT: Brachytherapy; ⑯ LCR: Locoregional Control Rate; ⑰ IMRT: Intensity-Modulated Radiotherapy; ⑱ SIB: Simultaneous Integrated Boost; ⑲ MTD: Maximum Tolerated Dose; ⑳ LRFS: Locoregional Recurrence-Free Survival; ㉑ RRFS: Regional Recurrence-Free Survival; ㉒ DSS: Disease-Specific Survival; ㉓ European Organisation for Research and Treatment of Cancer.

## Data Availability

No new data were created or analyzed in this study. Data sharing is not applicable to this article.

## Abbreviations

The following abbreviations are used in this manuscript:

CC	Cervical Cancer
HPV	Human Papillomavirus
LMICs	Low- and Middle-Income Countries
RT	Radiotherapy
LACC	Locally Advanced Cervical Cancer
CCRT	Concurrent Chemoradiotherapy
CFRT	Conventional Fractionated Radiotherapy
IMRT	Intensity-Modulated Radiotherapy
VMAT	Volumetric Modulated Arc Therapy
HFRT	Hypofractionated Radiotherapy
MHRT	Moderately Hypofractionated Radiotherapy
SBRT	Stereotactic Body Radiation Therapy
LQ Model	Linear-Quadratic Model
α/β Ratio	Alpha/Beta Ratio
EQD2	Equivalent Biological Dose in 2 Gy Fractions
OTT	Overall Treatment time
LCR	Local Control Rate
OS	Overall Survival
CTRI	Clinical Trials Registry—India
LNM	Lymph node Metastasis
HDR	High-Dose-Rate Brachytherapy
ICBT	Intracavitary Brachytherapy
GI	Gastrointestinal
GU	Genitourinary
DFS	Disease-Free Survival
SIB	Synchronous Integration Boost
IGABT	Image-Guided Adaptive Brachytherapy
BT	Brachytherapy
FIGO	International Federation of Gynecology and Obstetrics
CCR	Complete Clinical Response
RFS	Recurrence-Free Survival
NCT	National Clinical Trial
3D-CRT	Three-dimensional Conformal Radiation Therapy
QUANTEC	Quantitative Analysis of Normal Tissue Effects in the Clinic
OAR	Organ-at-Risk
2D-EBRT	Two-dimensional External Beam Radiotherapy
ORR	Overall Response rate
ART	Adaptive Radiotherapy
HDR-BT	High-Dose-Rate Brachytherapy
iCBCT	iterative Cone-beam Computed Tomography
CTCAE	Common Terminology Criteria for Adverse Events
RECIST	Response Evaluation Criteria in Solid Tumors
EORTC	European Organization for Research and Treatment of Cancer
PFS	Progression-Free Survival
CRR	Complete Response Rate
LPFS	locoregional Progression-Free Survival
CSS	Disease-Specific Survival
QOL	Quality of Life
CTC	Circulating Tumor Cell
MTD	Maximum Tolerated Dose
MFS	Metastasis-Free Survival
ABC-RT	Accelerated Brachytherapy combined with Hypofractionated Radiotherapy and Concurrent Chemotherapy
LRFS	Local Recurrence-Free Survival
RRFS	Regional Recurrence-Free Survival
HICs	High-Income Countries
DSS	Disease-Specific Survival
INCan	National Institute of Cancerology
RBE	Relative Biological Effectiveness
TIME	Tumor Immune Micr oenvironment
